# Foreign Bodies in the Rectum

**Published:** 1916-04

**Authors:** 


**Foreign Bodies in the Rectum.** Llewellin Eliot, Washington, Wash. Med. Annals, Jan. These bodies may have been swallowed, introduced through the anus, or may perforate through the male bladder or female vagina. Erotism is a large factor. King Edward II of England was murdered in 1327 by plunging a red hot iron into the rectum through a horn tube. (Note: It is explained that this method was employed as the assassins considered the person of royalty too sacred to be mutilated in any ordinary way). The Greeks punished an adulterer by introducing a peeled radish covered with hot ashes into the rectum. (Note: A somewhat analogous punishment in vogue among the Central American aborigines, for anyone who violated a virgin, was to twist a branched thorn in the urethra. If the victim recovered, there was no further punishment but, obviously, he usually died from urinary retention or septicaemia).

Among published reports of foreign bodies which have been swallowed some will be mentioned: needles, pins, bones, fruit seeds, large pieces of meat, gristle, false teeth, coins, forks, knives, mice, bits of glass and pieces of jewelry; of those formed in the intestines are: various enteroliths incident to bilious disorders and hardened feces; while among those introduced through the anus we find: bottles, pieces of wood, drinking glasses, snails, coins, jewelry, the frozen tail of a pig, and military despatches.

The necessaire, a thing common among the equipment of criminals, consists of a cylindrical box of wood, closed at one end with wax; it contains several pieces, sometimes as many as thirty, which fit into one another so as to form a file or a saw, the box making a convenient handle. Although this instrument is very imperfect, still with patience and application iron bars may be sawn through. A necessaire will ordinarily measure from five to seven inches, and while it looks innocent enough, it may cause the death of the person concealing it. Closmadeuc recorded an instance where one unfortunate had concealed the instrument, but it caused such a peritonitis that death resulted, and at autopsy it was found in the transverse colon. By some jugglery of imagination this instrument often receives his name.

The length of the sojourn of these bodies in the rectum may be a few hours, some days, sometimes several months, and occasionally some years.

It would require too much time to quote the many reports of foreign bodies removed from the rectum; let it suffice to make mention of a few of the more important and singular: Adler; the handle and the valve of a steam radiator pipe, 1½ inches in its smallest diameter, 2½ inches at the other end. Shields; a bar of silver 5½ inches long, 8½ inches in circumference, and weighing 30 ounces. Sevilla; a glass insulator used for telegraphic wire; abdominal section was required; death resulted. Truell removed a large stone by abdominal section; Warren; a catsup bottle 10 inches long, 9 inches in circumference, and refers to a case of a beet 7 inches long, 3½ inches in diameter; 3½ multiplied by 3.1416 makes 10.9956 inches in circumference; he refers also to a case of Walker, where a turnip 9½ inches in circumference was removed. Rhett Goode; the bulb of a 16-candlepower incandescent electric globe, 7½ inches in circumference, was pushed into the rectum for the relief of piles; the globe was broken in extraction, the rectum wounded and death resulted; in the discussion of this paper, C. W. Stiles spoke of a soldier who had regularly, for two or three months, introduced turkey heads into the rectum in order to obtain his discharge from the army; also of a specimen that had gone from laboratory to laboratory, without a solution of its identity, and was finally proved to be the fetus of a kitten that a man had introduced into his rectum. Stiles also spoke of a woman who had introduced fish entrails, and of another who had used those of a pigeon. Jenkins; a case where tramps had assaulted and robbed a man; not getting enough money from him, they took a turnip, hollowed out the lower end, put a potato in the hollow, and passed a string through them from end to end, then pushed the thing into his rectum. Pollak; a champagne flask 12½ inches long, 2¾ inches in diameter, 8-2/3 inches in circumference; had been in the rectum for 26 days. Dahlenkamp; a piece of oak bark, 4½ inches long, found 5 inches up the rectum, had been there for ten years. White; a ten-pin, 10 inches long, 2 inches in circumference. Adler; the bones of a fetus of extra-uterine pregnancy, and as some of our members may recall, P. J. Murphy reported a similar instance to this Society.

The author reports personal observations of faecal impaction, two of swallowed bone, and of a wicker covered cologne bottle, 10 inches long and 8 in circumference, accidentally introduced into the rectum instead of the vagina, where it had been employed for masturbation.

